# Computational Peptide Discovery with a Genetic Programming Approach

**DOI:** 10.21203/rs.3.rs-3307450/v1

**Published:** 2023-09-01

**Authors:** Nicolas Scalzitti, Iliya Miralavy, David E. Korenchan, Christian T. Farrar, Assaf A. Gilad, Wolfgang Banzhaf

**Affiliations:** 1 BEACON Center of Evolution in Action, Michigan State University, East Lansing, MI, USA; 2 Department of Computer Science and Engineering, Michigan State University, East Lansing, MI, USA; 3 Athinoula A. Martinos Center for Biomedical Imaging, Department of Radiology, Massachusetts General Hospital and Harvard Medical School, Boston, MA, USA; 4 Department of Chemical Engineering, Michigan State University, East Lansing, MI, USA; 5 Department of Radiology, Michigan State University, East Lansing, MI, USA

**Keywords:** Peptide discovery, Genetic programming, CEST MRI, contrast agent, regular expressions, Evolutionary algorithm

## Abstract

**Background:**

The development of peptides for therapeutic targets or biomarkers for disease diagnosis is a challenging task in protein engineering. Current approaches are tedious, often time-consuming and require complex laboratory data due to the vast search space. *In silico* methods can accelerate research and substantially reduce costs. Evolutionary algorithms are a promising approach for exploring large search spaces and facilitating the discovery of new peptides.

**Results:**

This study presents the development and use of a variant of the initial POET algorithm, called ${POET}_{Regex}$, which is based on genetic programming, where individuals are represented by a list of regular expressions. The program was trained on a small curated dataset and employed to predict new peptides that can improve the problem of sensitivity in detecting peptides through magnetic resonance imaging using chemical exchange saturation transfer (CEST). The resulting model achieves a performance gain of 20% over the initial POET variant and is able to predict a candidate peptide with a 58% performance increase compared to the gold-standard peptide.

**Conclusions:**

By combining the power of genetic programming with the flexibility of regular expressions, new potential peptide targets were identified to improve the sensitivity of detection by CEST. This approach provides a promising research direction for the efficient identification of peptides with therapeutic or diagnostic potential.

## Introduction

### Peptide-based Therapies and Diagnostics

Peptides are molecules composed of amino acids (AA) joined by peptide bonds. They are short sequences usually comprised of 2 to 50 AAs. Peptides are one of the cornerstones of living organisms and participate in many metabolic and physiologic activities, acting as hormones (*e.g.* insulin) [1], neurotransmitters [2], antimicrobial agents [3] or venoms [4, 5, 6]. Because of their intrinsic physicochemical properties (*e.g.* high selectivity and efficacy, low toxicity), peptides are a powerful target for therapeutic development [7, 8, 9, 10]. Indeed, since the first use of insulin over 100 years ago, peptides are being extensively studied as potential targets for various therapeutic applications such as cancer [11, 12, 13] or diabetes treatments [14]. They are also used to cater to a wide range of chronic [15] or rare diseases [16, 17], and have the potential to be used as vaccines [18]. More recently, they have been used to fight against Covid-19 [19]. In addition, peptides can serve as biomarkers for disease diagnostics. Indeed, human fluids, such as blood plasma, contain a wide range of proteins and peptides that represent a large source of physiologic information. Peptide biomarkers are used in different disease diagnostics such as cancer [20, 21], type II diabetes [22] or neurodegenerative disorders such as Alzheimer disease [23]. Peptides are also used in imaging diagnostics as positron emission tomography [24], single-photon emission computerized tomography [25] and chemical exchange saturation transfer (CEST) magnetic resonance imaging (MRI) [26, 27, 28].

### Protein Engineering

Because of their wide range of applications in therapies and diagnostics and their advantages over traditional drugs, peptides have tremendous potential in biomedical field. However, despite billions of years of evolution, the protein and peptide search space is not fully explored. Thus, the discovery and design for new peptides is a gargantuan task that researchers are trying to solve through two main approaches: i) rational design and ii) Directed Evolution (DE). In rational design, scientists use knowledge of a protein/peptide (*e.g.* crystalline structure) to optimize a new valuable target with desired functional and structural properties [29, 9]. DE is based on a model protein with similar function to the desired one, however, does not require more prior knowledge. This approach uses iterative mutagenesis and screening, which are the main operators to generate new targets guided by artificial evolution [30, 31, 32]. Unfortunately, these methods are not the Holy Grail for generating new therapeutic/diagnostic peptides, and some disadvantages slow down the research [33]. Indeed, these methods are time-consuming and costly. Moreover, necessary prior knowledge and wet lab experiments can be a limit. Finally, the search space is extremely complex, and the optimization trajectories could lead to local optima.

### Computer-aided Design of Peptides

To overcome these problems, researchers have started to use new computational methods generally based on machine learning (ML) and optimization techniques in the last decades. The advent of artificial intelligence (AI) allowed the development of new methods and tools to predict the structure or the function of proteins and peptides [34, 35]. Furthermore, Evolutionary Algorithms (EA) are widely used in the computational design of proteins and peptides [36, 37]. EAs are bioinspired metaheuristic optimization algorithms and are powerful tools to solve search and optimization problems [38]. One of the main advantages of EAs is the ability to explore a large search space [39]. Considering the creation of a peptide with 12 AAs, the search space is then 20^12^ possible targets (including only 20 classic AAs). Thus, EAs are very suitable to travel in this space and to discover new therapeutic/diagnostic peptides.

### Related Work

The journey of EA in protein design is relatively recent, with about 30 years of research. Many works focus on the prediction of the three-dimensional structure of proteins or their function, or on the motif discovery. In the 1990s Unger *et al.* developed an approach based on a genetic algorithm (GA) for protein folding simulations [40]. In 1995, Koza *et al.* exploited genetic programming (GP) to evolve motifs for the identification of the D-E-A-D box family of proteins and for the detection of the manganese superoxide dismutase family [41]. One year later, Yokobayashi *et al.* developed a method based on DE and a GA to generate new peptides with more efficient inhibitory activities. By carrying out artificial evolution, they obtained an improvement of more than 90% for some peptides [42]. Hu *et al.* proposed a GP method to identify patterns in protein sequence. They used a PROSITE [43] pattern representation, close to regular expressions (REs) for representing individuals [44]. Based on Hu’s works, Ross *et al.* used stochastic REs as a new representation language for protein sequences classification. A GP algorithm is then applied to evolve the stochastic REs and obtained promising results [45]. Heddad *et al.* also used a GP algorithm to generate and evolve RE-based classifiers. Their approach uses these classifiers to determine the nuclear localization of a protein [46]. In 2005, Seehuus *et al.* exploited a GP algorithm to discover patterns in biological sequences. They used linear GP to evolve individual represented by a REs. Their method has shown comparable results to those found in PROSITE [47]. In 2007, Yagi *et al.* proposed a new approach called ‘*in silico* panning’ for the selection of peptide inhibitors. They exploited a docking simulation associated with a GA to evolve target peptides. Interestingly, they showed the effectiveness of *in silico* evolution combined with experimental data [48]. In 2011, Becerra *et al.* proposed a procedure to predict the three-dimensional structure of proteins. Their strategy is based on a multi-objective parallel *ab initio* algorithm. They used the NSGA-II multi-objective GA to optimize the energetic contributions of the protein [49]. Yousef *et al.* combined a GA and protein free energy minimization calculations for the prediction of the three-dimensional structure of proteins [50]. Recently, Yoshida *et al.* used a combination of a GA and an *in vitro* evaluation. The individuals are potential antimicrobial peptides and the fitness function is the wet lab test. With this *in silico*-*in vitro* approach, they obtained promising results and identified 44 new antimicrobial peptides with 160-fold efficiency [51]. In the same year, Porto *et al.* published an approach based on a GA to design a guava antimicrobial peptide (one of the first plant-based peptides) [52].

### Development of the ${POET}_{Regex}$ Tool

In this context, we developed a new computational approach based on GP for new peptide discovery. Our method is based on the initial version of Protein Optimization Engineering Tool (POET) [53, 54], with a modification in the representation of individuals. Instead of a list of motifs of contiguous AAs, individuals are represented as a list of regular expressions (REs) to identify motifs with more flexibility. Indeed, the specific characteristics of the elements (operators) comprising the syntax of REs enable them to effectively identify motifs through the combination of these elements. These motifs are associated with weights calculated during a specific training step (see Materials and Methods). Therefore, the main objectives of this study are to evolve protein-function models based on REs, identify representative motifs and generate new peptides for a specific problem. Evolving REs with a GP algorithm is a method capable of exploring a huge search space and to find good solutions. REs are powerful tool and are widely used in computational evolutionary research for pattern or motif discovery, and text extraction [55, 56, 57, 58, 59, 60, 61, 62, 63].

As proof of concept, we apply our method to address the problem of the sensitivity of peptides to be detected by MRI with the chemical exchange saturation transfer. CEST is a MRI contrast approach where exchangeable protons are saturated by radiofrequency irradiation [64]. This saturation is then transferred with water protons and the signal can be detected. Contrast detection by CEST has a great potential for clinical imaging [65]. Initially, poly-L-lysine (composed from 12 lysine residues) was used as CEST contrast agent to paved the way for the search of new sensitive agents and is now considered the gold standard [66]. Since peptides are interesting agents for CEST contrast [27], we used our method to discover new peptides that provide high CEST contrast.

## Results

### Data

**Figure 1:**
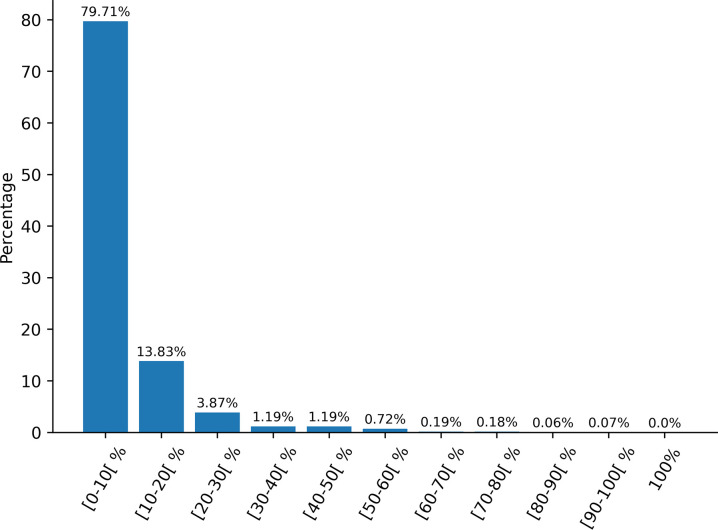
Average pairwise sequence percent identity in the dataset.

In order to verify that the dataset contains unique data and that certain sequences are not over-represented, we performed a pairwise sequence similarity calculation on the whole dataset (Figure 1). The results were averaged and grouped into sets of ten percent and show that most of the sequences (~80%) share less than 10% identity, demonstrating that the dataset used is heterogeneous. Only a very small portion of the data (1.22%) have more than 50% identity. Moreover, there are no completely identical sequences.

**Figure 2:**
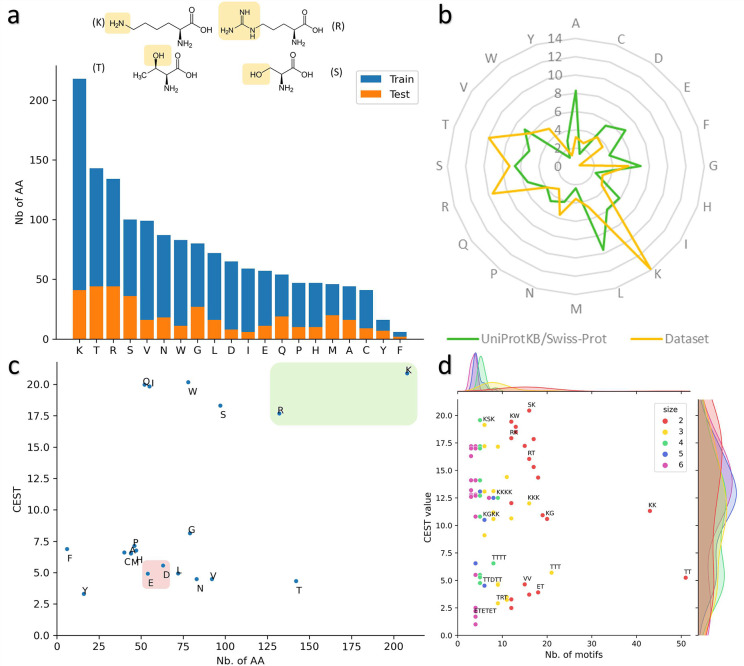
**a)** Frequency of occurrence of each AA in both training (blue) and test (orange) sets. Molecules represent the four most prevalent AA in the training set, and hydroxyl or amine groups are highlighted. **b)** Comparison of the frequency of each AA in our dataset (yellow) and in the UniProtKB/Swiss-Prot database (green). The different values represent the percentage of occurrence. **c)** Potential CEST value associated with each AA by occurrence method. The green box represents positively charged AA and the red box represents negatively charged AA. **d)** Frequency of the 20 most observed motifs (size 2 to 6) in the training set with the associated CEST value.

We then proceeded to conduct a more detailed analysis of the dataset. Initially, we examined the frequency of occurrence of each AA in both the training and test sets, as illustrated in Figure 2a. Our observations indicated that lysine (K), threonine (T), arginine (R), and serine (S) were among the most commonly occurring AAs in both sets. These AAs are polar and possess either hydroxyl, amine or guanidine (3 amines) groups, which facilitate the magnetic polarization of water in their environment. In addition, K and R are positively charged, making them compatible with the CEST mechanism. These properties allow them to accept protons and to be soluble in water. Tyrosine (Y) and phenylalanine (F) are the least frequent AAs in the dataset. These AAs are relatively uncommon in natural proteins accounting for only 2.92% and 3.86%, respectively. Their hydrophobic and aromatic nature may explain their low occurrence in the dataset.

Upon comparing the frequency of AAs occurrence in our dataset with UniProtKB/Swiss-Prot (release 2023 01) (Figure 2b), we noted an over-representation of K, R, T, and tryptophan (W), which is consistent with our earlier results. Interestingly, while W is infrequently present in UniProtKB/Swiss-Prot proteins (at a frequency of 1.1%), it is present in our dataset at a frequency exceeding 5%, indicating that it could play a significant role. Previous studies have demonstrated that the indole ring NH protons of W contribute to CEST contrast at approximately 5.5 ppm [67]. However, the CEST values in our dataset were measured at 3.6 ppm, which may suggest that the amide group in the backbone which would resonate at 3.6 ppm and may be responsible for generating a signal at this specific frequency. The AAs that are underrepresented in our dataset are alanine (A), phenylalanine (F), isoleucine (I), and leucine (L), which are non-polar and hydrophobic, lacking amine or hydroxyl groups in their side chains, as well as glutamic acid (E) and aspartic acid (D), which are negatively charged. Because a peptide with high CEST is required to be soluble in water, it is not surprising to find fewer hydrophobic AAs in the dataset.

Next, we conducted an analysis of the impact each AA may have during the evolutionary process (Figure 2c). Using the ‘occurrence’ method described in the Materials and Methods section, we calculated the potential CEST value associated with each AA. Our results indicate that AAs with the highest associated CEST values are K, R, S, Q, I, and W, while T, F, Y and the two negatively charged AAs, E and D, have relatively low CEST values. However, it should be noted that these values may vary depending on the context in which the AA is present, as CEST values are measured on a global peptide sequence. For instance, while W has a potential CEST value of approximately 20, the ‘*KWR*’ motif has a CEST value of 17.27, and the peptides containing this motif have CEST values of 18.46 and 16.08. This initial analysis has allowed us to identify two groups of AAs. Specifically, we have observed that six AAs have a CEST value >15, which could potentially guide the evolutionary process towards the production of REs with significant weight. Conversely, the other AAs have a CEST value <10.

Subsequently, we conducted a similar analysis on the 20 most prevalent patterns (ranging in size from 2 to 6) in the training set, as depicted in Figure 2d. As anticipated, motifs of size 2 and 3 dominate in the MDB. Notably, the most frequently occurring motifs consist of K or T. Although present, the divide between motifs with a CEST value greater than 10 and those below 10 is less noticeable. Many motifs with a high CEST contain K, R, and S, whereas motifs with low CEST values comprise T, E and D. These findings are consistent with our earlier analyses and provide valuable insights for scrutinizing the performance of the evolutionary algorithm.

### Management of the Number of Regular Expressions

**Figure 3:**
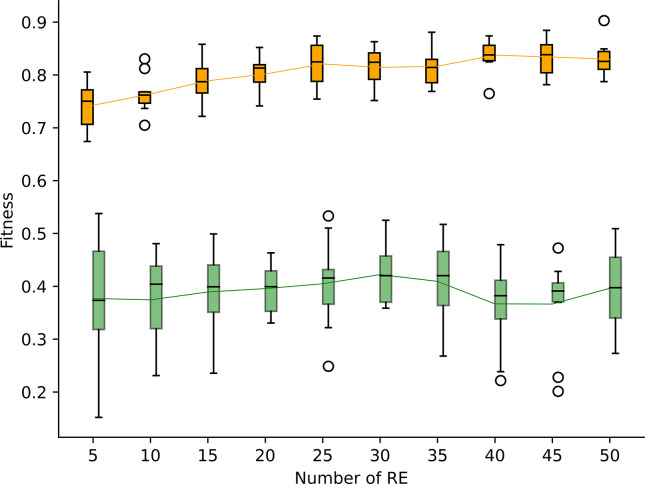
Performance of the models with a number of REs ranging from 5 to 50. The orange boxplots represent the results obtained on the training set, while the green boxplots represent the results on the test set. Within each boxplot, the black horizontal line represents the median, while the green and orange solid lines represent the mean values.

We conducted a series of 10 experiments, wherein we varied the number of REs within the range of 5 to 50. The primary objective of this experiment was to determine whether our model tended to do overfitting or underfitting. Overfitting occurs when the model closely matches the input data, thereby hindering its ability to generalize the data adequately. Conversely, underfitting transpires when the model is not precise enough and fails to generalize the data. The results of this analysis are graphically depicted in Figure 3.

We observe that the performance on the training set tends to increase from 5 to 50 REs. However, the performance on the test set increases progressively until 30 REs and after that point, the performances decrease on average. A low number of REs such as 5 or 10, restricts the ability of the models to generalize the data accurately. We observe that the average results for the training set are 0.742 and 0.764 for 5 and 10 REs respectively. On the contrary, a high number of REs can lead to overfitting, which is observed with the models having a number of REs higher than 30. Indeed, the average performances on the training set are high (>0.8) while the performances on the test set decrease. Therefore, in the next experiments we kept a maximum limit of 30 REs per individual.

### Comparison between ${POET}_{Regex}$ and ${POET}_{Rdm}$

**Figure 4:**
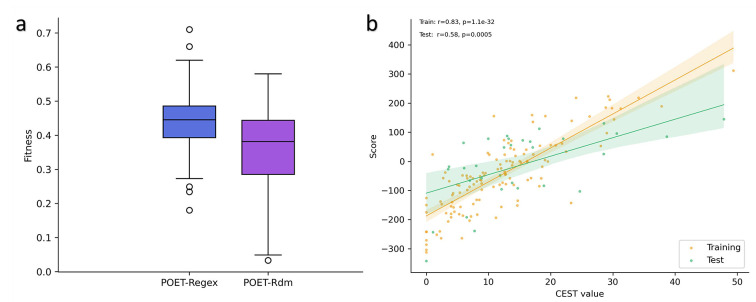
**a)** Comparison of ${POET}_{Regex}$ (blue) and ${POET}_{Rdm}$ (purple) models on the test set. **b)** Performance of the best ${POET}_{Rdm}$ model on the training set (orange) and the test set (green). The translucent bands around the regression line represent the confidence interval for the regression estimate.

To confirm the effectiveness of the training step during the evolutionary process, we conducted 50 independent experiments using identical parameters to ${POET}_{Regex}$ (Table 4 in Material and Methods section), but without the adjusting step of the RE weights. Each RE weight was randomly defined during initialization and could only be modified through a mutation with a probability of p=0.1. The results of these experiments are presented in Figure 4a and called ${POET}_{Rdm}$.

As expected, the results of the experiments with random weights (${POET}_{Rdm}$) are lower than the results of the experiments with the training step (${POET}_{Regex}$). A paired t-test was performed and confirmed that the results are statistically significant (p-value=1.07e^−3^). Indeed, the average fitness value obtained on the test set with ${POET}_{Rdm}$ is 0.359 compared to ${POET}_{Regex}$ which is 0.443. The results of ${POET}_{Regex}$ are about 23% (+0.084) higher than the experiments with ${POET}_{Rdm}$. Among the ${POET}_{Rdm}$ models, the best model (Figure 4b) has a fitness value of 0.58 (with p-value=5.04e^−4^) on the test set. This fitness value is 0.13 (~22%) lower than the best model achieved using ${POET}_{Regex}$. These results confirm the importance and efficiency of the training step during the execution of the algorithm.

### Best ${POET}_{Regex}$ Model

**Figure 5:**
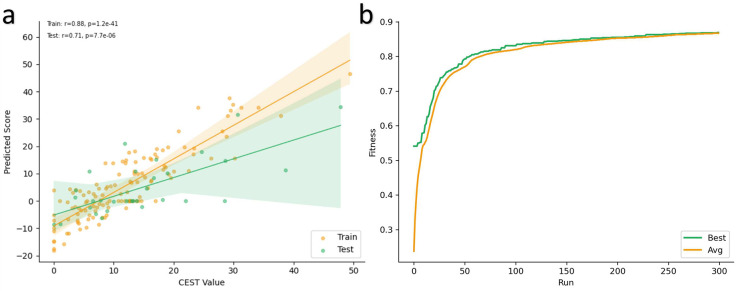
**a)** Performance of the best ${POET}_{Regex}$ model on the training set (orange) and on the test set (green). The strong correlation means that the algorithm has converged to a good solution. The translucent bands around the regression line represent the confidence interval for the regression estimate. **b)** Evolution of the fitness value during the evolutionary process. The green curve represents the fitness value of the best individual and the orange curve represents the fitness value of the entire population.

Out of all the experiments, the best ${POET}_{Regex}$ model (Additional file 2) exhibited interesting results with a strong correlation of 0.88 (p-value=1.2e^−41^) on the training set and 0.71 (p-value=7.7e^−6^) on the test set (Figure 5a). A correlation exceeding 0.5 indicates a highly positive correlation. Furthermore, a p-value below 0.05 indicates that the results are statistically significant. As shown in Figure 5b, the fitness values of the best individual and for the entire population continue to improve until around 100 generations and then tends to stabilize. This means that the algorithm converges to a good solution. It is interesting to note that this model comprises 29 rules, consisting of a combination of REs (80%) and contiguous motifs (20%). For instance, the ‘*KL*’ motif is one of the contiguous motifs with a weight of 3.397. Finally, these results confirm that our GP algorithm is capable of evolving protein-function models adapted to the CEST problem. Consequently, the algorithm is effective in identifying motifs that can enhance the CEST signal.

### Comparison between ${POET}_{Regex}$ and initial POET

In order to evaluate the efficiency of adding REs to build protein-function models, we conducted 100 experiments using the initial version of POET as a baseline for comparison. The same training set was used for both POET models and ${POET}_{Regex}$ models, and the same test set was also used. The default parameters utilized in [53] were employed throughout the experiments.

**Figure 6:**
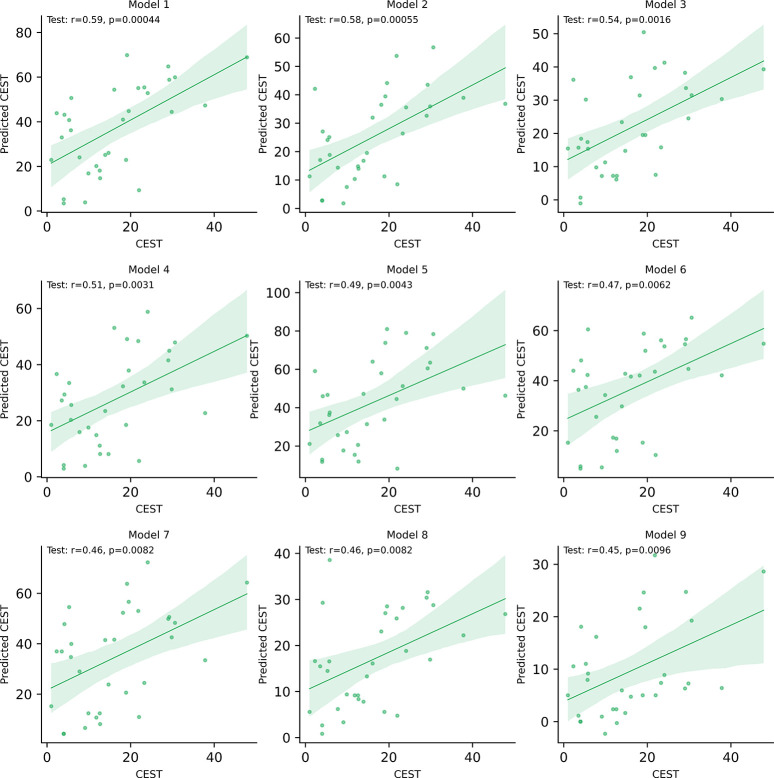
The 9 best POET models. Each dot represents a datapoint with a true CEST value associated with a predicted CEST value. The green line represents the regression line and the translucent bands around the regression line represent the confidence interval for the regression estimate.

On average, POET exhibits a correlation of 0.292 and a p-value of 0.205. Some models drastically reduce the average because the evolutionary process was unable to find a good solution, or the algorithm converged too fast and got stuck in a local maximum. Therefore, we focus only on the 9 best models to take advantage of the best results. The average correlation of the top 9 POET model is 0.504 (average p-value of 4.68e^−3^), which is very close to the performance obtained by ${POET}_{Regex}$. Figure 6 displays the results of the top 9 POET models. Model 1 obtains the best performance with a correlation coefficient of 0.59 and a p-value of 4.4e^−4^, meaning the result is statistically significant. These results are interesting and demonstrate the potential of the initial version of POET. However, the best ${POET}_{Regex}$ model performed better than POET and indicates that REs adds flexibility that POET does not have and improves the learning and prediction potential. The power and accuracy of the REs allowed ${POET}_{Regex}$ to perform better with an increase of the performance of 20% (+0.12).

### Peptide Predictions

The best ${POET}_{Regex}$ model (Additional file 2) and the standard encoding with 20 AAs are used to predict new peptides that possess the potential for a high CEST value. In this context, the higher the predicted score, the higher the CEST value of the predicted peptide should be. We performed 3 experiments with varying numbers of cycles (1000, 100 and 10 cycles) during a *in silico* DE process. This approach replaces the DE screening step by selecting peptides with a potentially high CEST value and drastically reduces experimental time and costs. The results for peptides with the highest predicted score (top 1) and peptides with a high predicted score and high hydrophilicity (best) for each experiment can be found in Table 1, while all predictions are available in Additional file 1: Table S3. It is important to highlight that the higher the number of cycles, the more the peptides generated will be similar and converge towards an identical solution. Conversely, a limited number of cycles results in less accurate predictions, but it allows for broader exploration and the generation of original peptides. Determining the optimal number of cycles is therefore a key point in DE.

**Figure 7:**
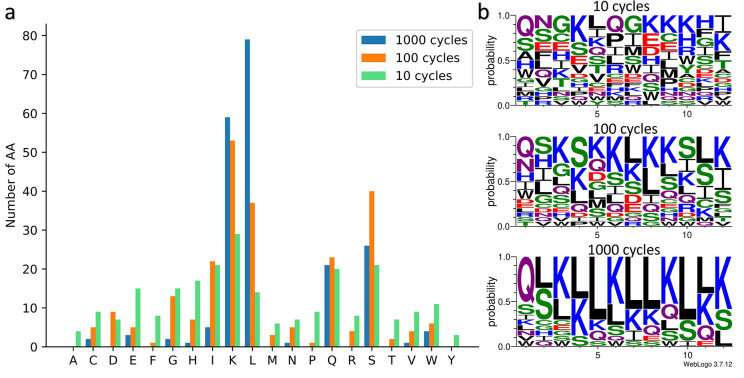
**a)** Number of AAs present in the predicted peptides in the 3 types of DE experiments: 1000 (blue), 100 (orange) and 10 (green) cycles. **b)** Sequence logos highlighting the probability of each AA at a given position, for the 3 experiments. As the number of cycles increases, the predicted peptides are more similar with high rates of lysine and leucine. The polar AAs are in green, the neutral in purple, the positively charged in blue, the negatively charged in red and the hydrophobic in black.

Next, we proceeded to analyze the AA composition of the predicted peptides. The results are illustrated in Figure 7a. As expected, peptides generated after 1000 cycles exhibited a homogeneous AA composition and achieved high predicted scores (>90). In contrast, peptides generated after 100 and 10 cycles displayed a more heterogeneous AA composition and obtained lower scores (about 70–80 for 100 cycles and 40–50 for 10 cycles). The sequence logos in Figure 7b generated with the WebLogo 3 tool [68], highlight the probability of each AA for a given position. With an increasing number of cycles, the presence of Q, L, S, and K becomes more prominent, confirming the tendency to converge towards similar peptides with a homogeneous AA composition.

We have also observed an important presence of isoleucine in predicted peptides in experiments involving 100 and 10 cycles (Additional file 1: Table S4). The abundance of lysine, glutamine, and serine in the predicted peptides is consistent with our initial analysis of the dataset. Lysine is a positively charged AA and plays a crucial role in detecting CEST signals. Glutamine and serine are non-charged polar AAs with amide and hydroxyl groups that facilitate proton exchange with water molecules. Hence, we expected to find these AAs in the predicted peptides. Conversely, we anticipated a high presence of arginine and tryptophan, given their abundance in the dataset. However, the peptides predicted for 10, 100, and 1000 cycles only contained 1.6%, 3.3%, and 0% arginine, respectively, and 4.5%, 2.5% and 1.6% of tryptophan. Interestingly, we observed a significant occurrence of leucine in the predicted peptides, with percentages of 5.83% for 10 cycles, 15.42% for 100 cycles, and 32.92% for 1000 cycles. This is interesting because leucine is not very abundant in the dataset. Leucine is also a hydrophobic AA, which contradicts the preference for hydrophilic and soluble peptides in CEST experiments. However, leucine plays a key role in protein structure folding and has a strong tendency to form alpha helices while maintaining their stability. Consequently, we used the ColabFold tool [69] based on the AlphaFold2 model [34] to perform 3D structure predictions of the leucine-rich predicted peptides. The results presented in Additional file 1: Figure S1 demonstrate that the predicted patterns tend to form alpha helices. Thus, the model can identify leucine-rich motifs that play a significant role in the formation of specific secondary structures such as the alpha helix. In this manner, the GP algorithm has produced original results. Despite our initial expectations of observing a substantial number of arginine, threonine, and tryptophan, it found and favored glutamine, leucine, and isoleucine. This suggests that the algorithm was capable of discovering motifs that contribute to the function and/or structure of the predicted peptides.

We identify the main motifs present in the predicted peptides for the three types of experiments. As anticipated, these motifs primarily consist of the residues K, L, Q, S, and I. In the peptides predicted after 1000 cycles, the main motifs involve lysine and leucine, such as *LK* (45), *KL* (38), *LLK* (28) or *LKLL* (17). However, there are also motifs that incorporate other AA, such as *LQS* (10) or *SLK* (16). In experiments involving fewer than 100 and 10 cycles, motifs such as *QS*, *GS*, *SI*, *SL*, or *SLK*, *LKS*, *IKK*, *LQS*, *QSL* are observed. These results confirm the ability of our algorithm to extract valuable information from the data and leverage it to generate peptides with potentially significant CEST values.

### Validation of Predictions

The protein-function models generated by ${POET}_{Regex}$ are used to predict novel peptides that have the potential to enhance CEST contrast. In order to validate the reliability of our approach, we selected the top 3 peptides with higher hydrophilicity and high score from each DE experiment (10, 100 and 1000 cycles) and assessed their performance under real wet-lab conditions.

**Figure 8:**
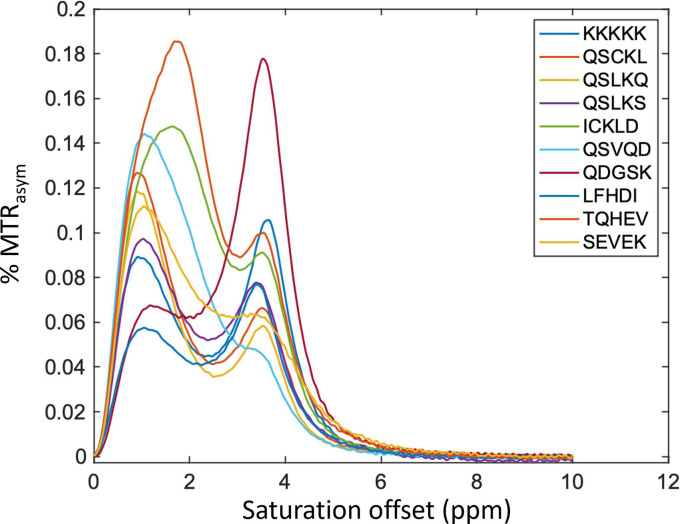
${MTR}_{asym}$ plot of nine peptides and the gold standard peptide (K12) measured by MRI.

The 9 peptides were synthesized and the magnetization transfer ratio asymmetry (${MTR}_{asym}$), a measure for CEST contrast, was obtained using MRI. The ${MTR}_{asym}$ was normalized to the molar concentration of the peptide (Additional file 3: Table S1) and plotted as a function of the saturation frequency offset (Figure 8). Since the ${POET}_{Regex}$ was trained from the ${MTR}_{asym}$ contrast at 3.6 ppm, the MRI results was presented in Table 2 for ${MTR}_{asym}$ at 3.6 ppm. These data were also normalized relative to the gold standard K12 peptide.

It is interesting to note that the results obtained from both the 1000-cycle and 10-cycle experiments do not demonstrate convincing results, showing an average ${MTR}_{asym}$ of 6.47 (1000 cycles) and 7.67 (10 cycles). These results are probably due to either too many or too few cycles, leading to the generation of homogenous or too diverse peptides. For instance, in the 1000-cycle experiments, 66% of the peptides consisted of QSLK or KLKK motifs, while no dominant motif was identified in the peptides from the 10-cycle experiments. Conversely, among the 3 peptides in experiment with 100 cycles, the peptide QDGSKKSLKSCK (QDGSK brown line in Figure 8) generated ${MTR}_{asym}$ 58% greater than the gold standard peptide K12 at 3.6ppm. An interesting observation is that this peptide contains only 25% K residues, which is important for increasing the diversity of the AA composition of genetically encoded reporters [28]. QDGSKKSLKSCK is also unique compared to other peptides since it has a distinct peak at 3.6 ppm, resulting from the amide exchangeable protons, with little or no contribution from amine or guanidine exchangeable protons resonating between 1.8–2.0 ppm.

These results suggest that ${POET}_{Regex}$ can be used to narrow the search space and identify good candidate. However, it is important to note that ${POET}_{Regex}$ can be trained at several different times to obtain better performance. By incorporating the experimental peptide data into the dataset and refining the hyperparameters of the model, we can enhance the performances of ${POET}_{Regex}$, similar to the improvements observed in the initial POET version [53], where 8 epochs were used.

## Discussion

Peptides have emerged as highly promising candidates as therapeutic targets, biomarkers for disease diagnostics and medical imaging [9], particularly as MRI CEST contrast agents [27]. They have several advantages, including high specificity, biodegradability, minimal tissue accumulation, and low toxicity. However, they also have certain disadvantages, such as low oral bioavailability, limited membrane permeability, low solubility, and expensive and time-consuming synthesis [70]. Due to the challenges associated with generating new peptides through traditional experimental methods, several computational approaches have emerged to aid in the peptide discovery. Among these, deep learning (DL) algorithms, including large language models [71], have recently gained prominence and show significant potential in different fields, such as synthetic biology and protein engineering [72, 73]. However, these models require a large amount of data. For instance, ProGen [73] used a training dataset consisting of 280 million proteins. In the realm of synthetic biology, the scarcity of experimental and curated data often remains a problem, as the available datasets are generally too small, making it impractical to employ DL methods [74]. To overcome these limitations, alternative computational approaches, such as evolutionary algorithms, can be employed to identify candidate peptides more efficiently.

The discovery of new peptides with potential for therapeutic or diagnostic purposes is a complex task that involves exploring a large search space. Unfortunately, exhaustive exploration of this space is not possible with current methods. Indeed, this is a NP-hard optimization problem, and the ratio between functional and non-functional proteins is heavily in favor of non-functional ones. Consequently, we have employed a heuristic approach based on a GP algorithm, which has proven effective in navigating complex search spaces where other methods may not perform as well. The GP algorithm, inspired from evolutionary mechanisms, is capable of finding satisfactory solutions to a given problem without prior knowledge, making it suitable for situations where solutions are not easily defined. To enhance the capabilities of our method, we coupled the GP algorithm with the power of REs, to identify motifs in peptide sequences. While REs are robust tools, the manual tuning of REs can be a time-consuming, tedious, and error-prone process [75]. Therefore, developing a method that can automatically generate REs for a given problem is a challenge, but has the potential to facilitate peptide discovery and protein engineering.

In this study, we introduce ${POET}_{Regex}$, a new tool designed to discover new peptides for a given problem. To illustrate the feasibility of our method, we used ${POET}_{Regex}$ to predict peptides with increased sensitivity detected by CEST. ${POET}_{Regex}$ used GP to optimize protein-function models, represented by a list of REs. While the initial version of POET relied on a list of motifs of contiguous AAs, restricting the peptide discovery, this new version incorporates two significant enhancements. Firstly, it leverages the flexibility of RE to identify specific motifs, enabling a more expansive exploration of the search space. Secondly, the training step takes advantage of high-quality data generated in the laboratory to adjust RE weights, eliminating the random assignment approach. This step enhances the identification of motifs that are crucial to the specific problem. In addition, ${POET}_{Regex}$ exhibits the ability to train on small datasets, which distinguishes it from DL models. Previous studies have demonstrated the potential of combining algorithms with limited datasets to achieve interesting results [76, 77]. Finally, the utilization of RE ultimately ensures the complete transparency of the model. Indeed, while DL models are often regarded as black boxes that are challenging to interpret, our model, despite relying on initially complex RE, is fully explainable. This ensures comprehension during prediction, detection of biases, user confidence, and continuous model improvement. Consequently, this contributes to a more ethical and effective utilization of AI.

By combining GP with RE, we were able to achieve a 20% improvement in performance compared to the previous version of POET. While this combination proves to be an interesting and efficient solution, it is important to note that motif search can be limited by the complexity of the motifs. Indeed, our approach relies on constructing a MDB that consists of a set of motifs found in the training dataset. Some motifs may be more complex and less prevalent, which can impact model training and subsequent predictions. Therefore, the construction of the MDB from the data is a key point of our study, and it is likely that increasing the amount of data could improve the performance of our strategy. Moreover, the ability of an RE to extract motifs is related to its length, which corresponds to the depth of the binary tree. To generate suitable REs, we adopted the ramped half and half method, which allows for the creation of a heterogeneous population of trees with varying depths. This approach strikes a balance between the complexity of RE and its ability to generalize to new data. However, using shallow trees can result in small RE that may lead to overfitting. These small REs can only extract specific motifs, limiting flexibility and hindering the ability of the model to generalize. Conversely, excessively deep trees produce long REs that may lead to underfitting. Long REs has the potential to extract a wide range of motifs, losing specificity, especially if there are numerous alternative choice operators (|). Additionally, large REs may contain regions that are not utilized during the training step but could play a significant role during the prediction step. These instances of ”false positives” can introduce bias into the predictions. Hence, it is crucial to select an appropriate RE size and number of REs, to avoid overfitting, underfitting, and the propagation of non-exploited regions. Another key to the success of the model is its ability to generalize data, and this requires that REs be heterogeneous, *i.e.* they do not extract the same motifs. Furthermore, it is worth noting that the best ${POET}_{Regex}$ model (Additional file 2) primarily consists of variable-sized REs but also incorporates fixed-size motifs. These results, combined with the high correlation coefficient obtained during the training step, indicate that our algorithm can extract (from the data) essential and specific motifs to address the problem at hand, while introducing the flexibility needed to generate innovative solutions. Finally, enhancing the performance of the ${POET}_{Regex}$ program could be achieved by incorporating additional data and refining the algorithm itself. Optimizing the parameters of an evolutionary algorithm is a complex task in its own right.

After the evolutionary process, we used the best ${POET}_{Regex}$ model to generate new peptides with higher sensitivity of detection by CEST using a DE method, which is a powerful tool for protein engineering [30]. Traditional DE involves generating a population of individuals with similar characteristics to the desired outcome, but this approach often gets stuck in local optima due to the similarity of starting points. Additionally, it relies on performing mutations and wet lab evaluations (screening step), which can be time-consuming and expensive. By employing a model like ${POET}_{Regex}$, we replace the screening step. The model can extract motifs of interest (or the inverse if the score is negative) to select the most promising peptides for the next generation. This broader coverage of the search space increases the likelihood of escaping local optima. Furthermore, the extrapolation capacity of our model enables it to generate original peptide sequences. Indeed, the peptides designed by ${POET}_{Regex}$ were found to be rich in lysine, serine, glutamine, leucine and isoleucine, whereas the input data contained a high number of lysine, serine, threonine and arginine and few glutamine, leucine and isoleucine. This indicates that the model favored motifs with a higher frequency of amino acids lysine and leucine while avoiding motifs containing arginine and threonine. The significant presence of lysine is consistent due to its amine group and positive charge, but the inclusion of leucine is original as it is a hydrophobic AA. Predictions of the 3D structure of leucine-rich peptides suggest that this residue plays an important role in the three-dimensional conformation of the peptide. The generation of secondary structures contributes to improved thermal stability of proteins [78]. In the future, by combining peptides obtained through the evolutionary algorithm with proteins, it may be possible to achieve a stable structure without compromising the potential for enhanced detection through CEST contrast. A similar approach has been successfully employed in the generation of *de novo* biosensors for CEST MRI by coupling proteins with peptides exhibiting high CEST potential [28].

Finally, nine peptides generated by ${POET}_{Regex}$ were carefully selected, synthesized, and their ${MTR}_{asym}$ values were calculated. Among these peptides, only one demonstrated intriguing results, displaying a remarkable increase of over 58% compared to the gold standard K12. Although there was no apparent correlation between ${POET}_{Regex}$ prediction scores and experimental outcomes (probably due to the limited number of peptides synthesized), this discrepancy might be attributed to the impact of either excessive or insufficient cycles during the DE. Nevertheless, we successfully identified a promising candidate peptide (QDGSKKSLKSCK) that exhibited interesting characteristics when compared to genetically encoded reporters. These results demonstrates that our method is capable of evolving protein-function models to extract motifs that align with a given problem even in the presence of initial constraints. Moreover, it has the capacity to reduce the search space and leverage a more comprehensive range of amino acids. It is essential to emphasize that these results were obtained from a single epoch, implying that we can further improve the performance of the model and enhance the sensitivity of generated peptides by improving the dataset with additional experimental data.

## Conclusions

The development of the ${POET}_{Regex}$ tool represents a significant advance in the field of protein engineering. This study highlights the effectiveness of combining genetic programming with regular expressions to efficiently explore a vast search space and generate new peptides, which could lead to the development of new therapeutic targets and biomarkers. Although our study focused on the use of ${POET}_{Regex}$ to improve the sensitivity of CEST-based imaging, the program could also be applied to other areas of protein engineering. The flexibility of RE provides a precise, explainable, and targeted approach for identifying specific motifs, making ${POET}_{Regex}$ applicable beyond the scope of our study. Considering the increasing prominence of personalized medicine and the expanding utilization of peptides in the pharmaceutical market, we firmly believe that *in silico* approaches like ${POET}_{Regex}$ can play a crucial role in accelerating the discovery of new peptide targets.

## Materials and Methods

This section describes the data used, the GP algorithm combined with REs and the different steps to obtain predicted peptides.

### Datasets

Having good quality and curated data is a fundamental requirement to train an accurate model. Unfortunately, high-quality data is rare, and databases often contain a significant amount of erroneous data [79]. Therefore, the curated dataset used in this study is mainly based on data from wet lab experiments [53, 54]. The dataset contains 158 sequences of peptides ranging from 10 to 13 AAs in length. The 20 standard AAs are used and the CEST values were measured at 3.6 ppm. Then, two datasets are generated to train and test the models. The training set contains 127 ( 80%) randomly drawn sequences, and the test set the remaining 31 ( 20%) sequences. The whole dataset is available as Additional file 1: Table S1.

### Motif Database Construction

To train the model by adjusting the weight of each RE, the algorithm uses a list of motifs extracted from the training dataset. Extracted motifs are the atomic units of information in the evolutionary process. These motifs are recovered using a sliding window of a size varying from 2 to 6 AAs which is applied on each sequence (single AA are also extracted). To determine whether a motif should be favored or not, it is assigned a class based on the CEST value of the sequence where the motif is extracted. Class 0 is chosen if the motif has a negative impact on the peptide results (<threshold) and class 1 if the motif has a positive impact on the peptide (>threshold). The threshold is defined based on the experiment. In this study, the threshold was set at 12.5, which corresponds to the CEST value of the poly-L-lysine peptide (K12) in the dataset, which is the gold-standard peptide [80, 64]. Since the training sequences are associated with a CEST value, it is possible to associate a CEST value with each extracted motif. However, the same motif may be present in several training sequences with different CEST values. To address this issue, a strategy called ‘occurrence’ is implemented to associate a CEST value with a motif. To do this, the number of motifs present in both class 1 and class 0 sequences is extracted. The class exhibiting the highest number of motifs is chosen and the average value is retrieved. The final motif database (MDB) contains 5360 motifs from 2 to 6 AAs, each associated with a CEST value and a class.

### Sequence Identity

In order to verify that there is no over-representation of sequences with identical motifs or identical sequences in the dataset, a sequence similarity search was performed on all sequences in the dataset to calculate the percent identity per pair according to the following formula:

$$\%Identity=\left(\frac{Nb\;\text{Identical}\;\text{AA}\;}{\;\text{Sequence}\;\text{Length}\;}\right)\times 100$$

### Regular Expressions

A RE is a sequence of characters (including operators and variables) that describe, according to a precise syntax, a search pattern in a target text. The operators used for this study are presented in Table 3. REs are implemented with the re library of python version 2.2.1.

### Genetic Programming

**Figure 9:**
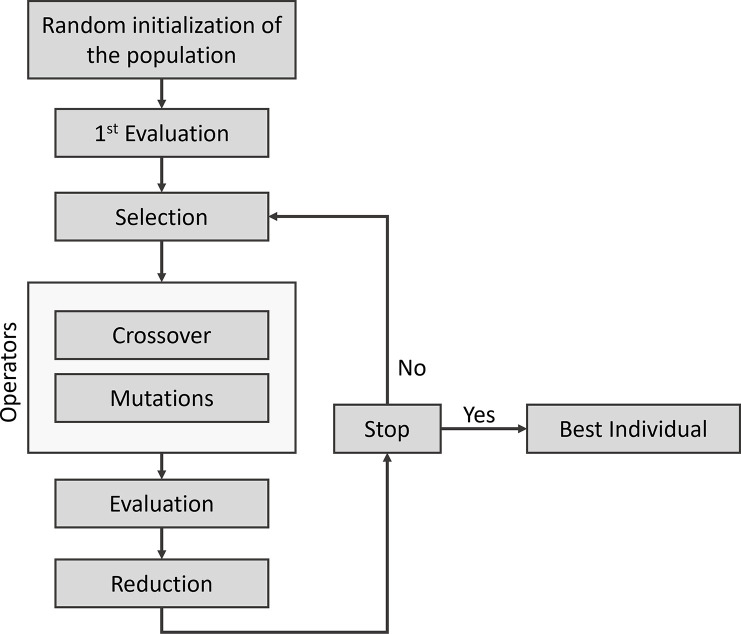
Classical evolutionary cycle of a GP algorithm

GP algorithms [81, 82] are powerful evolutionary computing techniques, a branch of AI and are widely used in different fields, such as engineering or bioinformatics [83]. GP is a stochastic algorithm (an extension of GA) inspired by the Darwinian evolution concepts and is useful for automatically solving complex optimization problems [81]. This type of algorithm is designed to explore a large search space and generate potential solutions through evolutionary mechanisms: selection, recombination (or crossover) and mutations. The solutions represent individuals in the population P that GP will evolve. In each iteration, all individuals are evaluated based on a fitness function to obtain a fitness score, which is used to rank each individual according to their ability to solve the problem. Two evolutionary operators are then classically applied: crossover and mutations. During the crossover, a selection of two parents is made and a subpart of parent 1 is exchanged with a subpart of parent 2. This operation generates two new offspring with a mix of the characteristics of their parents. Consequently, population size is increasing. The mutation operators are then applied. Depending on the problem and the representation of the individual, these operators can vary. Typically, these mutation operators involve the addition, deletion or substitution of an element of the target individual. Finally, new individuals are evaluated using the fitness function and in the reduction step only the S best individuals (with S the initial size of the population) are selected to be included in the population P+1 to continue their evolutionary journey in a new run. The evolutionary cycle ceases when a stop condition is reached, such as time, number of runs, or the algorithm finding a good solution. Figure 9 illustrates the evolutionary cycle of GP used in this study.

### Representation of Individuals

Unlike GA, where individuals are fixed-length strings, individuals in GP are represented by computational programs, usually as a tree structure (an acyclic network consisting of nodes connected by edges) [82] or a linear shape (such as instructions) [84]. GP manipulates these programs with different arithmetic operations, however the tree structure also allows the use of the syntax of REs.

**Figure 10:**
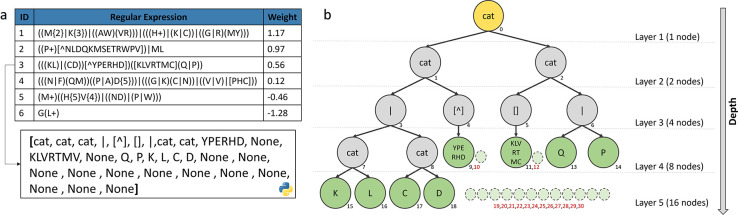
**a)** Representation of an individual (a protein-function model) as a list of rules with 3 columns (ID, regular expression pattern and weight). An example (RE3) is represented as a built-in list structure in Python, where a parent node i has 2 children: (i*2)+1 and (i*2)+2. **b)** Representation of RE3 as a binary tree. The yellow node is the root, grey nodes are the internal nodes and green nodes are the leaves. The small dotted nodes with red numbers are unexpressed nodes represented by ‘None’.

In our method, an individual is a protein-function model represented by a list of rules, with each rule being composed of an ID, a RE and a weight (Figure 10a). Each RE is represented by a binary tree implemented as a list where node i is the parent, and node (i*2)+1 and (i*2)+2 are children (Figure 10b). Each internal node represents an operator, and each leaf (or terminal node) represents a variable. The maximal depth of a tree is 6, which prevents having REs that are too long and time-consuming to evaluate. REs are randomly generated using the ramped half-and-half strategy [85] to create a population with heterogeneous individuals. Initially, individuals have a list of rules between 1 to 8 RE with a weight equal to 0.

### Evolutionary Operators

Three main evolutionary operators are used in this study: i) selection ii) crossover iii) mutation. The next paragraphs describe these operators in more detail.

#### i) Selection

The selection operator plays a key role in evolution by determining which individuals will proceed to the subsequent steps of evolution (crossover and mutations). The individuals selected for the crossover are referred to as parents. One commonly used selection method in GP is tournament selection [86]. In classical tournament selection, a random sample of k individuals (which represents the size of the tournament) is chosen from the population with replacement. The best individual in the tournament is then selected to become a parent. The tournament size influences the selection pressure: a higher value of k reduces the likelihood of selecting a bad individual, whereas a lower value of k increases the chances of selecting a bad individual. In this study, a tournament size of k=5 has been chosen.

#### ii) Crossover

**Figure 11:**
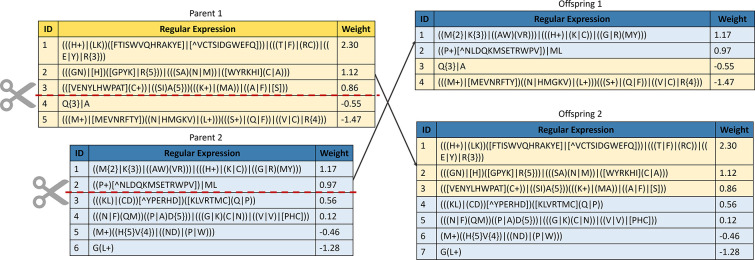
Representation of the one-point crossover. A subpart of parent 1 is merged with a subpart of parent 2 to produce an offspring.

The crossover operator involves the combination of a subpart of both parents to generate offspring. A one-point strategy is used, wherein a point in the parent is selected and cut to form two subparts (A and B). This process is repeated with the second parent. The next step involves exchanging part B of parent 1 with part A of parent 2 to create two offspring with a mix of elements from both parents. While the crossover operator provides diversity and can preserve important features, it may converge to a local maximum during the evolutionary process [87]. These characteristics make the crossover operator a widely used method for generating offspring with desirable traits in evolutionary algorithms. Figure 11 illustrates the one-point crossover operator used in this study.

#### iii) Mutations

**Figure 12:**
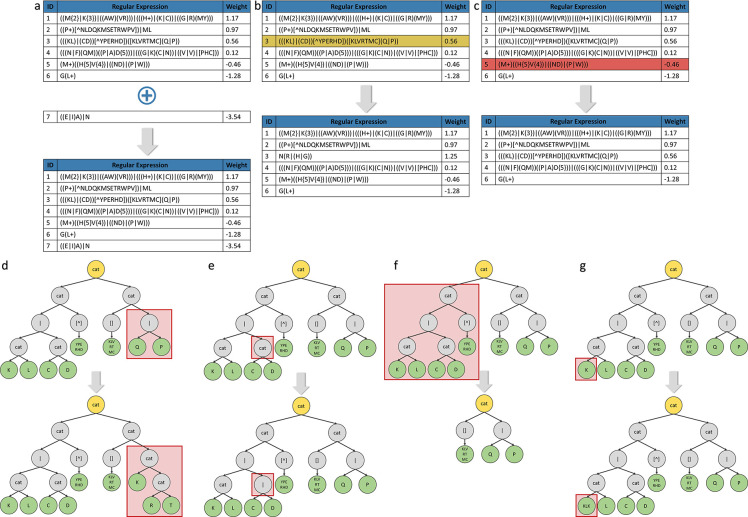
Representation of each type of mutation. **a)** Addition of a new rule in the list of rules. **b)** Replacement of a rule by a new rule. **c)** Deletion of an existing rule in the list of rules. **d)** Replacement of a branch of the tree. **e)** Inversion of a node. **f)** Deletion of a subtree. **g)** Add one or more AAs to a leaf.

Mutations allow to explore the search space by inducing changes in an individual, which increases the diversity of the population. This study implements two groups of mutations, with the first group targeting the individual as a whole and the second group targeting specific REs. Each individual or RE has a mutation rate of 10%. Group I contains 3 types of mutations:

- Addition of a new rule (Figure 12a), if the number of rules of the individual does not exceed the maximum threshold of the number of rules allowed, then a new rule is randomly generated and added to the list of rules of the individual.
- Replacement of an existing rule (Figure 12b), an existing rule is randomly selected and replaced by a new generated rule.
- Deletion of an existing rule (Figure 12c), if the list of rules of an individual has at least two or more rules, then an existing rule is randomly selected and removed from the rule list.

Group II contains 4 types of mutations, only impacting RE:

- Replacement of a branch of the tree (Figure 12d), randomly selects a branch of the tree (subtree) and replaces it with a new randomly generated one (its depth can vary).
- Inversion of a node (Figure 12e), randomly selects a node and changes its value. For example, cat (∅) becomes or (|), including bracket ([]) becomes excluding bracket ([ˆ]). If the node contains an AA, it is replaced by another random AA, and if the node is the value contained in the curly braces, then the value is replaced by another one.
- Deletion of a subtree (Figure 12f), randomly selects a subtree and deletes it.
- Addition of new AA in a leaf (Figure 12g), adds 1 to 4 new random AAs in the leaf, to create a specific motif.

Figure 12 illustrates the different mutation operators used in this study.

### Evaluation Function

Each individual is assigned a fitness value (or score) during the evaluation step. This fitness value reflects the degree of adaptation to the problem, with higher scores indicating better adaptation. In this study, the evaluation function chosen is the Pearson correlation coefficient. This coefficient ranges from −1 (indicating a strong negative linear correlation) to 1 (representing a strong positive linear correlation), while a value of 0 signifies no correlation. To compute the fitness value of an individual, each RE of its rule list is i) trained (*i.e.* its weight is adjusted) and ii) evaluated on a dataset. These steps are parallelized on 20 CPU cores to expedite the execution speed.

#### i) Training Step

The weights of the RE are initialized to 0 at the beginning of the algorithm. In each generation, the fitness value is computed to determine the best individuals. The first step consists in adjusting the weight of each RE based on the motif database (MDB) constructed from the training dataset. Each RE is tested against each training sequence, and the resulting matches (motifs) are extracted. For instance, if the RE ‘*[PNYIQ]K+*’ is applied to the sequence APVPKKPRLL, it will identify the motif ‘PKK’. This motif is associated with a CEST value in the MDB. The score of the RE on this sequence corresponds to the CEST value multiplied by the size of the motif. If the CEST value is greater than the threshold (here is 12.5) then we add the value to the final score, otherwise we subtract it. The final weight of the RE is the sum of all scores obtained on each training sequence, as shown in the following equation:

$$\;\text{Final\_Weight}\;=(\sum_{i=1}^{n}\;\;\{\begin{array}{ll} +CEST_{\text{motif\_i}\;}\times Size_{\text{motif\_i}\;}, & \;\text{if}\;\text{CEST}\;\geq T\\ -CEST_{\text{motif\_i}\;}\times Size_{\text{motif\_i}\;}, & \;\text{if}\;\text{CEST}\;<T \end{array})$$

with n the number of extracted motifs in the training sequences and T the threshold.

#### ii) Evaluation Step

After the training step, individuals are evaluated on each sequence in the training set. The *predicted_score* is the sum of all weights of the REs that match in the sequence. This *predicted_score* is then combined with the true CEST value of the sequence evaluated by the individual and the Pearson correlation coefficient is used to calculate the strength of the linear relationship between the *True_CEST* and the *predicted_score*. To prevent overfitting, cross-validation is performed using the k-fold method with k=6. The evaluation step is therefore performed on data from the training set (r_train) (as the training step) but also on an evaluation set (r_eval) which corresponds to 16% of the initial training set. Consequently, the fitness of the individual corresponds to the average of r_train, while the average of r_eval is used to assess the behavior of the algorithm and identify any signs of overfitting.

### Elitism

Before each mutation and crossover step, the best individual (elite) is extracted and automatically included in the next generation with no change, to prevent the performance of the algorithm from decreasing.

### Peptide Prediction

Once the algorithm has reached a stopping condition, such as the maximum number of generations or the fitness value is plateauing, meaning that the algorithm has reached a local optimum, the best individual can be used as a model for generating new peptides. Therefore, a higher predicted score should imply a higher CEST value for the predicted peptide.

*In silico* directed evolution (DE) coupled with the best ${POET}_{Regex}$ model, is employed for the prediction of new peptides. This approach has already been successfully applied in previous study [42, 88, 53]. Three DE experiments were conducted with different cycle numbers (10, 100, and 1000 cycles). Insufficient cycles could result in heterogeneous peptides and hinder algorithm convergence, while an high number of cycles may lead converged results and homogeneous peptides. A library of 1000 peptides is generated randomly at the beginning of each experiment and the peptide sequences then undergo three steps: mutagenesis, evaluation and selection. The mutagenesis step consists of introducing a random mutation (substitution of an AA) to generate new variants in order to increase fitness. The evaluation step employs a trained model, replacing the long, tedious and often expensive wet-lab screening process. Each peptide is evaluated using the best ${POET}_{Regex}$ model, which provides a score correlated to the presumed CEST value. If the fitness of the mutated peptide exceeds that of the initial peptide, the mutated peptide will subsequently replace the initial peptide and be selected for the next cycle. In the context of the identification of peptide with high CEST contrast, a filter is implemented to exclusively select hydrophilic peptides at the end of the evolutionary process. This filter calculates the sum of the hydrophobicity values for each AA (from [89], Additional file 1: Table S2). If the sum is greater than zero, the peptide is selected. Conversely, if the sum is equal to or less than zero, the peptide is eliminated from consideration and classified as non-soluble. Finally, from the remaining peptides, the top 20 are extracted, as they are considered to have the highest potential CEST value among the selected hydrophilic peptides.

### Peptide Synthesis and Preparation

Each peptide generated by the ${POET}_{Regex}$ model is synthesized by Genscript USA Inc. (Piscataway, NJ). Peptides were prepared by dissolving 4–5 mg of peptide in 600 *μ*L of PBS, then titrating the solution to pH 7.25–7.30 (measured using a pH electrode calibrated between pH 7 and 10 at room temperature) using using 0.1 M HCl and 0.1 M NaOH. Each solution was then pipetted into a separate 5 mm NMR tube.

### CEST NMR Measurements

The CEST data were acquired on a 14.1 T vertical-bore Bruker Avance III HD NMR spectrometer with the sample temperature set to 37°C. For each sample, the probe was tuned and matched as soon as the sample temperature was reached and stable, then the sample was shimmed manually on the water proton resonance and the 90° pulse length was calibrated by finding the 360° zero-crossing and dividing by four. The spin-lattice relaxation time constant ($T_{1}$) was measured for each sample using an inversion-recovery sequence modified to include a z-gradient pulse at 5% of the maximum amplitude between the inversion and excitation pulses, to reduce radiation damping [90]. Z-spectra were obtained at least 40 minutes after the sample temperature probe reached stability, so that the sample had sufficient time to equilibrate. The CEST sequence was an ultrafast z-spectroscopy sequence [91] with the following parameters: 2048 acquired FID points, 42.6 kHz bandwidth, 32 scans, 10 s recovery delay between scans, 5% gradient applied during saturation and acquisition, pulse offset frequency set to be ~3250 Hz higher than water frequency, 5 s saturation pulse, saturation power varying from ~1.2–5.2 *μ*T with 10 powers measured per sample. Four dummy scans were performed in between each saturation power value. Each sample also included a reference scan ($S_{0}$, saturation power = 0 *μ*T) at the beginning and end of the z-spectroscopy; all samples showed little to no change between the two reference scans, indicating sample stability.

All z-spectral data were processed using custom-written MATLAB scripts, including scripts developed by the research group of Dr. Moritz Zaiss, publicly available on GitHub at https://github.com/cest-sources. Raw FID data were loaded into MATLAB, zero-filled by a factor of 16, Fourier transformed, and normalized by the first reference scan (saturation power = 0 *μ*T) to obtain z-spectra. The magnetization transfer ratio asymmetry $\left({MTR}_{asym}\right)$ was calculated using the z-spectral amplitudes at ±3.6 ppm and the following equation:

$$MTR_{asym}=\frac{S(-3.6ppm)-S(3.6ppm)}{S_{0}}$$

### Configuration

The experiments are performed on Michigan State University’s High Performance Computing Center computers. Each experiment utilized 20 CPU cores and 15 GB of RAM. A configuration file was employed to specify different hyper-parameters of the algorithm, which are summarized in Table 4. The scripts are implemented in python v3.10.6 and are available at the following link: https://gitlab.com/NicolasScalzitti/poet_regex.

## Figures and Tables

**Table 1:** Predicted peptides with highest predicted score (Top 1) and best predicted peptides with highest hydrophilicity and high score (Best), with 1000, 100 and 10 cycles during DE.

Cycles	Predicted peptide (Top 1)	Predicted score	Hydrophilicity

1000	ICKLLKLLKLLK	97.66	0.05
100	RLKSMQLKLDKL	82.83	3.25
10	QSCKYCQSLKFD	52.85	1.52
Cycles	Predicted peptide (Best)	Predicted score	Hydrophilicity

1000	QSLKQSIKKLKK	92.52	4.94
100	QDGSKKSLKSCK	74.55	5.37
10	SEVEKPFWEQDK	39.91	7.52

**Table 2:** Experimental results of the predicted peptides and the gold standard K12.

Peptides	${POET}_{Regex}$ score	MTR_asym (%)

**KKKKKKKKKKKK**	**N.A.**	**10.51**
QSCKLKKLQSLK	94.39	6.51
QSLKQSIKKLKK	92.52	5.72
QSLKSWIEKLKK	92.49	7.20
ICKLDKRIKKLK	80.52	8.96
QSVQDKLKKRII	77.18	4.36
QDGSKKSLKSCK	74.55	17.59
LFHDIEKQLKHA	43.79	7.01
TQHEVQSEKRGW	41.87	9.86
SEVEKPFWEQDK	39.91	6.14

**Table 3:** RE operators used in this study

Operator	Symbol	Description	Arity

Concatenation	Ø (invisible)	Concatenate two elements	2
Alternative choice (or)	|	Choice between two elements	2
Quantifier 1,n	+	Define a group present once or *n* time	1
Curly braces	{ }	Define the number of times the element is repeated	1
Bracket	[ ]	Define a list of choice between elements in the bracket	1
Excluding bracket	[^]	Define a list of choice between elements that are not in the bracket	1
Parenthesis	( )	Define a group	1

**Table 4:** Hyper-parameters used in ${POET}_{Regex}$ experiments

Hyper-parameter	Value

Population size	1000
Number of runs	300
Max RE	30
Crossover probability	0.9
Mutations probability	0.1
Tree depth	6

## Data Availability

The code and datasets analysed during the current study are available in the Gitlab repository, https://gitlab.com/NicolasScalzitti/poet_regex

## Abbreviations

POET: Protein Optimization Engineering Tool
CEST: Chemical Exchange Saturation Transfer
MRI: Magnetic Resonance Imaging
AA: Amino Acid
DE: Directed Evolution
ML: Machine Learning
DL: Deep Learning
AI: Artificial Intelligence
GA: Genetic Algorithm
GP: Genetic Programming
RE: Regular Expression
K12: poly-lysine peptide
MDB: Motif DataBase
EA: Evolutionary Algorithm
${MTR}_{asym}$: Magnetization Transfer Ratio asymmetry
